# Breaking the Taboo: Unveiling the Prevalence and Predictors of Female Sexual Dysfunction in Tunisia

**DOI:** 10.1192/j.eurpsy.2024.1598

**Published:** 2024-08-27

**Authors:** K. Razki, A. Larnaout, C. Najar, S. Ben Aissa, R. Lansari, W. Melki

**Affiliations:** Psychiatry department, razi hospital, manouba, Tunisia

## Abstract

**Introduction:**

Female sexuality is a complex and multifactorial domain that can be influenced by a variety of psychological, biological, relational, and sociocultural factors. However, sexual dysfunctions in women remain a taboo subject in many cultures and are often underestimated, underdiagnosed, and undertreated. In Tunisia, few studies have been conducted to assess the prevalence of sexual dysfunctions in women and their predictive factors.

**Objectives:**

to determine the prevalence of sexual dysfunctions in a group of Tunisian women and to identify the predictive factors of these dysfunctions.

**Methods:**

This is a cross-sectional, descriptive, and analytical study, over a period of three months, from September to December 2022, conducted online via a pre-established questionnaire to collect various sociodemographic data, personal history, psychoactive substance consumption, weight, and height. We used the Female Sexual Function Index (FSFI) scale to evaluate sexual functioning in participants. We recruited sexually active Tunisian women over 18 years of age who agreed to anonymously respond to the questionnaire. The form was disseminated on social networks, in groups that focus on women, with a rate of three publications per week.

**Results:**

We collected data from 90 women with a mean age of 35 ± 12.84 years.

More than half of our population (60%, n=54) had at least one sexual dysfunction.

The most common sexual dysfunctions reported were arousal disorders (31.3%), followed by desire disorders (26.8%) orgasm disorders (12.4%).

We found that several factors were significantly associated with sexual dysfunctions : Women over 45 years of âge (p<10^-3^), who are divorced (p=0,02), have a low socioeconomic status (p=0,04), and report having experienced traumatic romantic/sexual expériences (p<10^-3^) were found to have a higher prevalence of sexual dysfunctions.

According to our results, cannabis consumption had a negative impact on lubrication (p<10-3) and orgasm (p=0.003) among our study respondents. Personal psychiatric history also had a negative influence on arousal (p=0.02) and sexual satisfaction (p=0.01).

**Conclusions:**

By identifying sexual dysfunctions early and treating them effectively, we can improve the quality of life of those affected and avoid serious consequences on their physical and mental health. It is therefore crucial to promote a proactive approach to sexual health and encourage healthcare professionals to approach sexuality openly and comprehensively.

**Disclosure of Interest:**

None Declared

